# Acute kidney injury and in-hospital outcomes after transcatheter aortic valve replacement in patients without chronic kidney disease: insights from the national inpatient sample

**DOI:** 10.1186/s12872-024-04303-1

**Published:** 2024-12-19

**Authors:** Congyan Ye, Xueping Ma, Bo Shi, Rui Yan, Shizhe Fu, Kairu Wang, Ru Yan, Shaobin Jia, Shengping Yang, Guangzhi Cong

**Affiliations:** 1 Institute of Medical Sciences, General Hospital of Ningxia Medical University, Yinchuan, Ningxia China; 2 Institute of Cardiovascular Medicine, General Hospital of Ningxia Medical University, Ningxia, China; 3 Department of Cardiology, General Hospital of Ningxia Medical University, Ningxia Medical University, Ningxia, China

**Keywords:** Acute kidney injury, Propensity score matching, Transcatheter aortic valve replacement

## Abstract

**Background:**

Acute kidney injury (AKI) complicates transcatheter aortic valve replacement (TAVR), leading to higher mortality. The incidence and effects of AKI on clinical outcomes in patients undergoing TAVR without chronic kidney disease (CKD) are unclear. We aimed to determine the association between AKI and in-hospital outcomes in patients with TAVR using propensity score matching (PSM).

**Methods:**

Using International Classification of Diseases-10th Revision codes, we queried the National Inpatient Sample for TAVR performed between 2016 and 2021. Patients were divided into two groups according to perioperative AKI development. Patients with CKD or on permanent hemodialysis at baseline were excluded. We conducted 1:1 PSM to assemble a cohort of patients with similar baseline characteristics. Multivariate logistic regression was used to assess the association between AKI and in-hospital outcomes. Sensitivity analysis was conducted to evaluate the robustness of our inferences.

**Results:**

Of 47,372 unweighted patient admissions for TAVR, 1617 (3.41%) had a concomitant diagnosis of AKI. The incidence of AKI decreased from 4.82 to 3.18% from 2016 to 2021 (P-trend < 0.01). Before PSM, patients with AKI had a significantly higher rate of in-hospital mortality compared with those without AKI (6.12% vs. 0.48%, respectively; odds ratio [OR] 8.59, 95% confidence interval [CI] 6.32–11.68). Using the PSM algorithm, 1579 well-matched patients were included in each group. After PSM, an association was observed between patients with TAVR and concomitant AKI and a higher risk of in-hospital mortality (6.21% vs. 1.08%, respectively; OR 5.96; 95% CI 3.54–10.04). In subgroup analyses stratified according to age (≤ 80 and > 80 years), sex (male/female), and hypertension status, consistent associations were observed between AKI and the risk of in-hospital mortality. AKI patients were at higher risk for acute myocardial infarction (OR 1.78, 95% CI 1.35–2.34), major bleeding (OR 1.62, 95% CI 1.13–2.33), blood transfusion (OR 1.65, 95% CI 1.28–2.11), and cardiogenic shock (OR 3.73, 95% CI 2.77–5.01). No significant betweengroup differences were observed in stroke (*P* = 0.12).

**Conclusion:**

AKI was a strong predictor of in-hospital mortality in patients undergoing TAVR without CKD and was associated with higher post-procedure complication rates.

**Supplementary Information:**

The online version contains supplementary material available at 10.1186/s12872-024-04303-1.

## Background

A paradigm shift in the treatment of aortic stenosis (AS) has been witnessed in the last decade, with transcatheter aortic valve replacement (TAVR) emerging as an alternative option to surgical aortic valve replacement across all surgical risk strata. Despite advances in techniques and expertise, the occurrence of peri- and post-procedural complications continues to be significant [1].

Acute kidney injury (AKI) is a common post-TAVR complication, with the incidence ranging from 12 to 57% [2–4]. The pathogenesis of AKI after TAVR is multifactorial, including hemodynamic, inflammatory, and nephrotoxic factors [5–7]. While previous studies have suggested that AKI is a strong predictor of mortality at short- and long-term follow-up post-TAVR, these studies included a high percentage of patients with chronic kidney disease (CKD), which is an established risk factor for AKI [8–10]. With the expansion of the indications for TAVR to low-risk younger patients with longer life expectancy, overall comorbidity burden and procedural risk in patients are expected to reduce, potentially leading to a lower risk of AKI [11, 12].

There is a paucity of data describing AKI occurrence in patients undergoing TAVR who have normal baseline renal function, and its effects on mortality remain unknown. Moreover, research concerning the effect of AKI on the occurrence of adverse events such as acute myocardial infarction (AMI), stroke, cardiogenic shock (CS), bleeding, and the need for blood transfusions during hospital stay, which can be easily recognized and addressed promptly, is limited. Therefore, this study aimed to evaluate the potential association between AKI and clinical outcomes after TAVR in a contemporary cohort.

## Methods

### Study design and participants

In this observational, retrospective, nationwide, cohort study, hospitalizations for TAVR were identified using the National Inpatient Sample (NIS). The NIS is the largest nationwide inpatient healthcare database in the United States, covering > 7 million unweighted hospitalizations annually and comprising patient- and hospital‐level data from > 1000 hospitals, accounting for 20% of all hospitalizations in the United States [13]. The database is compiled annually, making it possible to analyze disease trends over time. The database contains detailed information on patient demographics, including ethnicity, age, sex, region, and other relevant characteristics, which allows for a comprehensive analysis of different population groups. The International Classification of Diseases, Tenth Revision Clinical Modification/Procedure Coding System (ICD-10-CM/PCS) is used to track diseases and procedures. Past research has shown that the NIS is a valuable tool for assessing the use trends and outcomes of new techniques and treatments in the field of aortic valve diseases, which gives confidence in its ability to provide meaningful insights for this study [14, 15]. The NIS database is de-identified and publicly available; therefore, institutional review board approval and patient-informed consent were not required for this study. This study, approved by the Ningxia Medical University Institutional Review Board, followed the Strengthening the Reporting of Observational Studies in Epidemiology (STROBE) reporting requirements [16]. The NIS database is open to the public and is available at www.hcup-us.ahrq.gov.

**Fig. 1:**
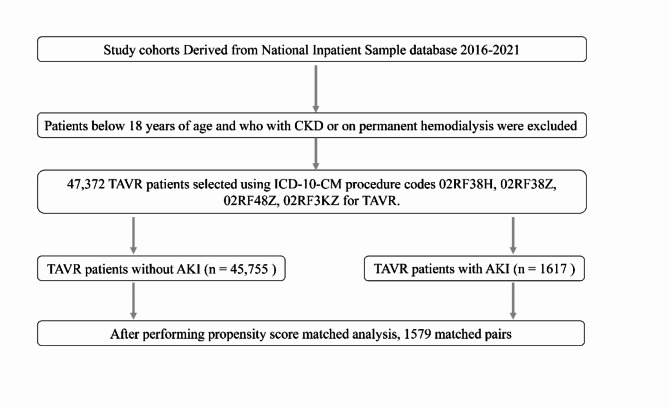
Study flowchart. TAVR indicates Transcatheter Aortic Valve Replacement; AKI, Acute kidney injury ; CKD, chronic kidney disease; ICD-­10, International Classification of Diseases, Tenth Revision

We retrospectively queried the database for the years 2016 to 2021. Hospital admissions for TAVR were identified using the following ICD-10-CM procedure codes: 02RF38H, 02RF38Z, 02RF48Z, and 02RF3KZ. All patients aged ≥ 18 years treated with TAVR were included. Exclusion criteria comprised patients with CKD (stage I and higher) or those on permanent hemodialysis at baseline (shown in Fig. 1). Using ICD-10 codes, AKI was defined as an acute renal failure with tubular, cortical, or medullary necrosis (ICD-10 code N17), post-procedural renal failure (ICD-10 code N99), or post-procedural complications of the genitourinary system (ICD-10 code N99.89). These administrative codes for AKI have a low sensitivity but a high specificity of approximately 99%, implying that our sample would have few false positives [17]. Validation studies have shown that 95% of validated cases have met the Kidney Disease Improving Global Outcomes (KDIGO) definition for AKI [18].

For each hospitalization, we described relevant clinical and socioeconomic features of patients, including demographic characteristics, insurance status, median household income based on the patient’s ZIP code; and hospital characteristics such as hospital location (rural, urban), bed size (small, medium, and large), and hospital teaching status. Clinical comorbidities were determined using the Elixhauser comorbidity index [19]. A list of ICD-10‐CM/PCS used to identify comorbidities is provided in Table S1.

### Outcomes

In-hospital outcomes were evaluated and compared between patients with and without AKI. The primary outcome of interest was all-cause in-hospital mortality. Secondary outcomes were AMI, major bleeding, the need for blood transfusion, stroke, and CS. Outcomes were abstracted using ICD-10 codes (Table S1).

### Statistical analysis

Due to the skewed nature of the NIS data, continuous variables are reported as mean ± standard deviation (SD) and interquartile range (IQR), depending on their distribution. Categorical variables are expressed as percentages. A chi-square test was used to compare categorical variables, while a Kruskal–Wallis test was used for continuous variables. Given the differences in baseline characteristics between eligible participants in the two groups (Table 1), propensity score matching (PSM) was used to identify a cohort of patients with similar baseline characteristics. The propensity score is a conditional probability of having a specific exposure (with or without AKI) based on a set of baseline measurable covariates. PSM was computed using a non-parsimonious multivariable logistic regression model, with patients with TAVR and AKI as the dependent variable and all the baseline characteristics outlined in Table 1 as covariates. PSM was performed using greedy closest neighbor matching, a 0.02 caliper, and a 1:1 match ratio. Standardized differences of < 10.0% for a given covariate indicate a relatively small imbalance [20].

**Table 1:** Baseline characteristics in the unmatched and propensity-score matched cohorts

	Before Matching				After Matching			
Variable	No AKI (*n* = 45,755)	AKI (*n* = 1617)	SMD	*P* Value	No AKI (*n* = 1579)	AKI (*n* = 1579)	SMD	*P* Value
Age, mean ± SD (years)	78.37 ± 8.49	78.62 ± 8.95	0.03	0.23	78.17 ± 9.02	78.70 ± 9.01	0.06	0.10
Sex			0.04	0.11			0.12	0.01
Male	24,283 (53.07%)	891 (55.10%)			795 (50.35%)	887 (56.17%)		
Female	21,472 (46.93%)	726 (44.90%)			784 (49.65%)	692 (43.83%)		
Race			0.11	0.01			0.05	0.64
White	40,948 (89.49%)	1391 (86.02%)			1357 (85.94%)	1353 (85.69%)		
Black	1285 (2.81%)	51 (3.15%)			61 (3.86%)	51 (3.23%)		
Hispanic	2014 (4.40%)	97 (6.00%)			93 (5.89%)	97 (6.14%)		
Other	1508 (3.30%)	78 (4.82%)			68 (4.31%)	78 (4.94%)		
Primary expected payer			0.11	0.01			0.07	0.37
Medicare/Medicaid	40,568(88.66%)	1420 (87.81%)			1387 (87.84%)	1383 (87.59%)		
Private insurance	4156 (9.08%)	141 (8.72%)			155 (9.82%)	141 (8.93%)		
Other	1031(2.25%)	46 (2.84%)			37 (2.34%)	55 (3.48%)		
Hospital characteristics								
Region			0.08	0.31			0.04	0.71
Northeast	13,466(29.43%)	444(27.46%)			391 (24.76%)	410 (25.97%)		
Midwest	18,746(40.97%)	659(40.75%)			686 (43.45%)	655 (41.48%)		
South	6222(13.60%)	240(14.84%)			239 (15.14%)	240 (15.20%)		
West	7321(16.00)	274(16.94%)			263 (16.66%)	274 (17.35%)		
Teaching			0.06	0.06			0.12	0.01
Rural	3331 (7.28%)	143(8.84%)			109 (6.90%)	141 (8.93%)		
Urban non-teaching	38,473 (84.08%)	1339 (82.81%)			1290 (81.70%)	1303 (82.52%)		
Urban teaching	3951 (8.64%)	135 (8.35%)			180 (11.40%)	135 (8.55%)		
Hospital bed size			0.14	0.01			0.43	0.01
Small	3179 (6.95%)	76 (4.70%)			249 (15.77%)	76 (4.81%)		
Medium	11,067(24.19%)	334 (20.66%)			397 (25.14%)	300 (19.00%)		
Large	31,509 (68.86%)	1207 (74.64%)			933 (59.09%)	1203 (76.19%)		
Weekend admission	865 (2.20%)	162 (8.32%)	0.28	0.01	161 (8.55%)	153 (8.13%)	0.02	0.64
Median household income			0.05	0.27			0.05	0.20
0–25th percentile	9299 (20.32%)	337 (20.84%)			370 (23.40%)	337 (21.30%)		
26–50th percentile	11,145 (24.36%)	422 (26.10%)			379(24.00%)	419 (26.50%)		
51–75th percentile	11,938 (26.09%)	396 (24.49%)			421 (26.70%)	396 (25.10%)		
76–100th percentile	13,373 (29.23%)	462 (28.57%)			409 (25.90%)	427 (27.00%)		
Length of stay	2.51 ± 2.56	7.81 ± 5.26	1.28	0.01	6.49 ± 5.62	7.55 ± 5.04	0.20	0.01
Cost of hospitalization	194631.41 ± 95340.65	258798.96 ± 116678.62	0.60	0.01	239302.01 ± 123445.97	259455.08 ± 117321.98	0.17	0.01
Comorbidities								
Smoking	19,016 (41.56%)	549 (33.95%)	0.16	0.01	694 (43.95%)	549 (34.77%)	0.19	0.01
Dyslipidemia	34,179 (74.70%)	1034 (63.95%)	0.23	0.01	1152 (72.96%)	1032 (65.36%)	0.17	0.01
Hypertension	40,026(87.48%)	1387 (85.78%)	0.05	0.04	1365 (86.45%)	1383 (87.59%)	0.03	0.37
Diabetes mellitus	14,616 (31.94%)	633 (39.15%)	0.15	0.01	479 (30.34%)	597 (37.81%)	0.16	0.01
Anemia	1698 (3.71%)	129 (7.98%)	0.18	0.01	103 (6.52%)	128 (8.11%)	0.06	0.09
Congestive heart failure	31,135 (68.05%)	1328 (82.13%)	0.26	0.01	1018 (64.47%)	1290 (81.70%)	0.40	0.01
Cardiac arrhythmia	23,089 (50.46%)	1022 (63.20%)	0.26	0.01	865 (54.78%)	1018 (64.47%)	0.20	0.01
Chronic pulmonary disease	11,402 (24.92%)	478 (29.56%)	0.10	0.01	410 (25.97%)	477 (30.21%)	0.09	0.01
Coagulopathy	3854 (8.42%)	338 (20.90%)	0.36	0.01	256 (16.21%)	300 (19.00%)	0.07	0.04
Liver disease	1466 (3.20%)	111 (6.86%)	0.17	0.01	82 (5.19%)	108 (6.84%)	0.07	0.05
Fluid and electrolyte disorders	3968 (8.67%)	565 (34.94%)	0.67	0.01	466 (29.51%)	561 (35.53%)	0.13	0.01
Other neurological disorders	1816 (3.97%)	152 (9.40%)	0.22	0.01	112 (7.09%)	151 (9.56%)	0.09	0.01
Peripheral vascular disease	8679 (18.97%)	375 (23.19%)	0.10	0.01	355 (22.48%)	375 (23.75%)	0.03	0.40
Hypothyroidism	8562 (18.71%)	285 (17.63%)	0.03	0.27	302 (19.13%)	285 (18.05%)	0.03	0.44
Obesity	9535 (20.84%)	326 (20.16%)	0.02	0.51	302 (19.13%)	326 (20.65%)	0.04	0.29
Weight loss	710 (1.55%)	101 (6.25%)	0.24	0.01	99 (6.27%)	101 (6.40%)	0.01	0.89
Depression	3782 (8.27%)	152 (9.40%)	0.04	0.10	153 (9.69%)	152 (9.63%)	0.00	0.95
Paralysis	339 (0.74%)	50 (3.09%)	0.17	0.01	33 (2.09%)	50 (3.17%)	0.07	0.06
Elix score index	4.82 ± 1.67	5.95 ± 1.75	0.66	0.01	5.36 ± 2.16	5.95 ± 1.76	0.31	0.01
History								
Prior MI	4875 (10.65%)	177 (10.95%)	0.01	0.71	149 (9.44%)	177 (11.21%)	0.06	0.10
Prior Stoke	5972 (13.05%)	208 (12.86%)	0.01	0.83	199 (12.60%)	208 (13.17%)	0.02	0.63
Prior PCI	9738 (21.28%)	259 (16.02%)	0.14	0.01	321 (20.33%)	259 (16.40%)	0.10	0.01
Prior CABG	6027 (13.17%)	220 (13.61%)	0.01	0.61	197 (12.48%)	218 (13.81%)	0.04	0.27

Note: Data are presented as mean ± SD for normally distributed continuous variables and median (Q1, Q3) for nonnormally distributed continuous variables. The chi-square test was used to compare categorical variables, whereas the Kruskal Wallis test was used for continuous variables

Abbreviations: PCI, percutaneous coronary intervention; CABG, coronary artery bypass graft; AKI, acute kidney injury; MI, myocardial infarction

Multivariable logistic regression was used to evaluate the effect of AKI on in­hospital outcomes, and data are reported as odds ratios (ORs) with 95% confidence intervals (CIs). To examine the robustness of our inferences, we performed several sensitivity analyses. We repeated the analyses stratified according to age (≤ 80 and > 80 years), sex (male/female), and hypertension status (determined using Elixhauser comorbidity measures). As kidneys are considered end-organs, a setting of CS can be the cause of AKI [21, 22]. We also tested whether the association would change if CS-concomitant patients were excluded.

Temporal trends in AKI incidence were examined using smooth curve fitting. Unweighted counts were used for all statistical analyses. Statistical significance was defined as two-tailed *P* < 0.05. Statistical analysis was performed using the R package (http://www.r-project. org) and EmpowerStats (http://www.empowerstats.com) software.

## Results

### Baseline characteristics

Of 47,372 identified patients who received TAVR and met our inclusion criteria, 1617 (3.41%) had a concomitant diagnosis of AKI (shown in Fig. 1). Baseline patient and hospital characteristics are shown in Table 1. Prior to PSM, no difference was observed between patients with and without AKI in terms of age (mean, 78.37 vs. 78.62 years; standardized mean difference [SMD] 0.03), female sex (46.93% vs. 44.90%, SMD 0.04), hypertension (87.48% vs. 85.78%, SMD 0.05), hypothyroidism (18.71% vs. 17.63%, SMD 0.03), or obesity (20.84% vs. 20.16%, SMD 0.02). Patients treated with TAVR with concomitant AKI were more often of non-White ethnicity (13.97% vs. 10.51%, SMD 0.11), had more comorbidities (5.95 vs. 4.82, SMD 0.66), and had been admitted to a large hospital (74.64% vs. 68.86%, SMD 0.14). After PSM, a sample of 3,158 patients (1,579 in each group) with well-matched baseline characteristics was identified (Table 1).

### In-hospital outcomes

**Fig. 2:**
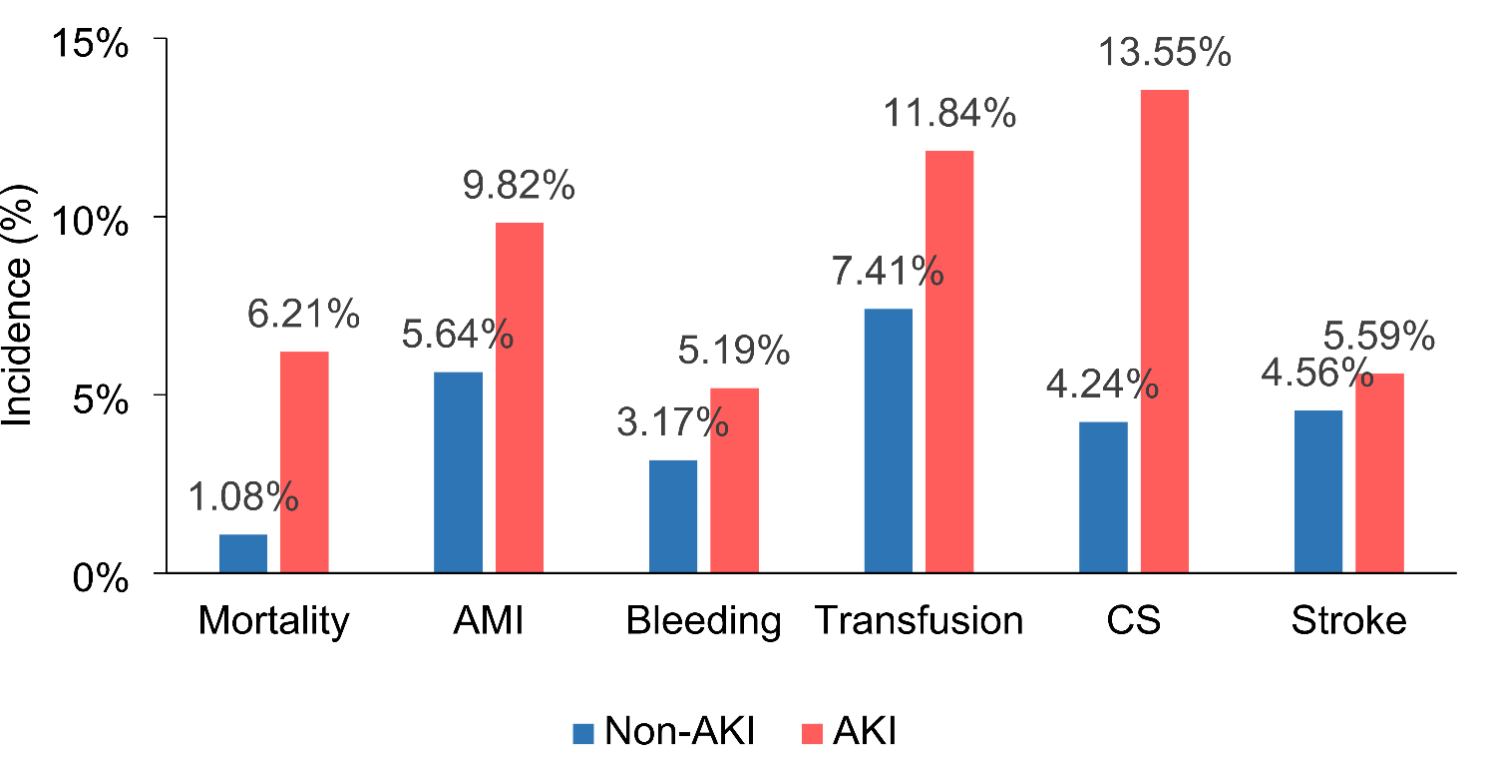
Comparison of in-­hospital mortality and incidence of complications between hospitalized AKI patients with and without AKI after propensity score matching. All p values are less than 0.01

After PSM, patients with AKI had higher in-hospital mortality (6.21% vs. 1.08%, OR 5.96, 95% CI 3.54–10.04), as well as more AMI events (9.82% vs. 5.64%, OR 1.78, 95% CI 1.35–2.34), bleeding (5.19% vs. 3.17%, OR 1.62, 95% CI 1.13–2.33), blood transfusions (11.84% vs. 7.41%, OR 1.65, 95% CI 1.28–2.11), and CS (13.55% vs. 4.24%, OR 3.73, 95% CI 2.77–5.01). There was no significant difference between the two groups in terms of stroke risk (5.95% vs., 4.56%, respectively; *P* = 0.07) (shown in Fig. 2; Table 2).

**Table 2:** In-Hospital outcomes of AKI Versus Non-AKI in the propensity-score matched cohort

	OR	95% CI	*P*-value
In-hospital mortality	5.96	(3.54, 10.04)	< 0.01
AMI	1.78	(1.35, 2.34)	< 0.01
Bleeding	1.62	(1.13, 2.33)	< 0.01
Blood transfusion	1.65	(1.28, 2.11)	< 0.01
CS	3.73	(2.77, 5.01)	< 0.01
Stroke	1.28	(0.94, 1.77)	0.12

Abbreviations: AMI, acute myocardial infarction; CS, cardiogenic shock

Subgroup analysis revealed that a TAVR hospitalization with AKI was consistently associated with high mortality risk across all subgroups. Unweighted ORs are shown in Table S2.

### Sensitivity analysis

**Fig. 3:**
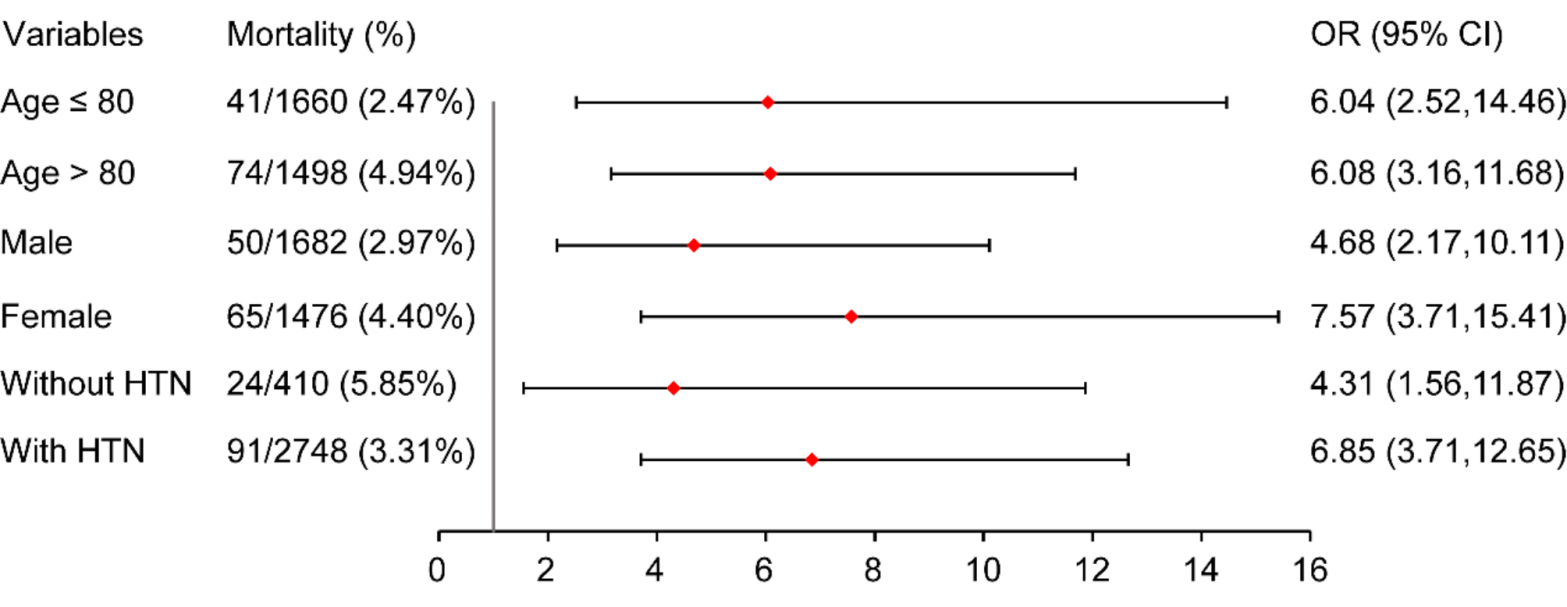
Forest plot of mortality rates according to pre-specified subgroups

Fig. 3 presents the outcomes of subgroup analyses after PSM. TAVR hospitalization with AKI was consistently associated with high mortality risk across all subgroups. After excluding 281 patients with CS, the risk effect of AKI was still significant, with a 4.45-fold increase (95% CI 2.32–8.57; *P* < 0.001) shown in the primary outcome (Table S3).

### Trends in AKI incidence and in-hospital death in patients with and without AKI

**Fig. 4:**
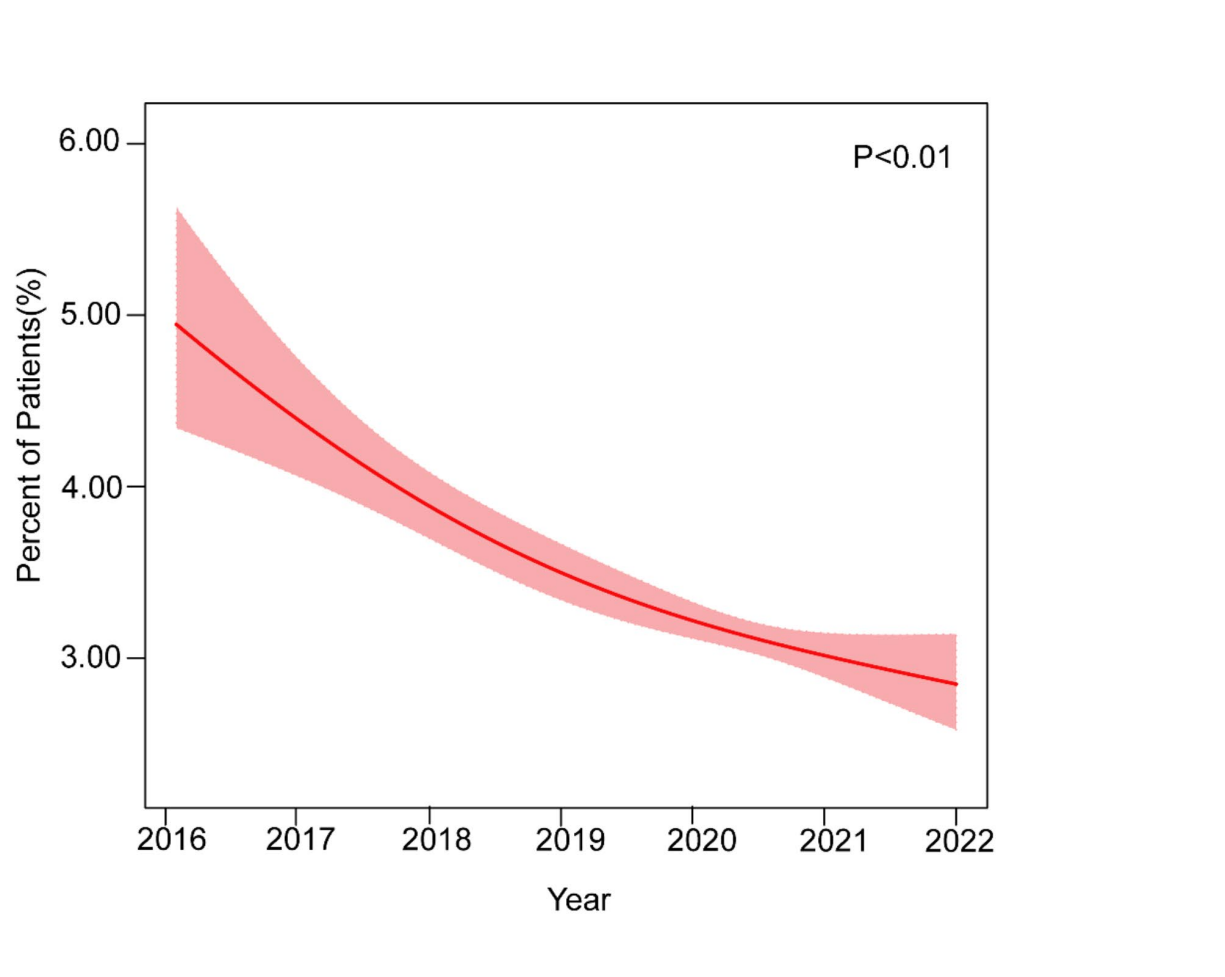
Trends in AKI incidence in patients with and without AKI

**Fig. 5:**
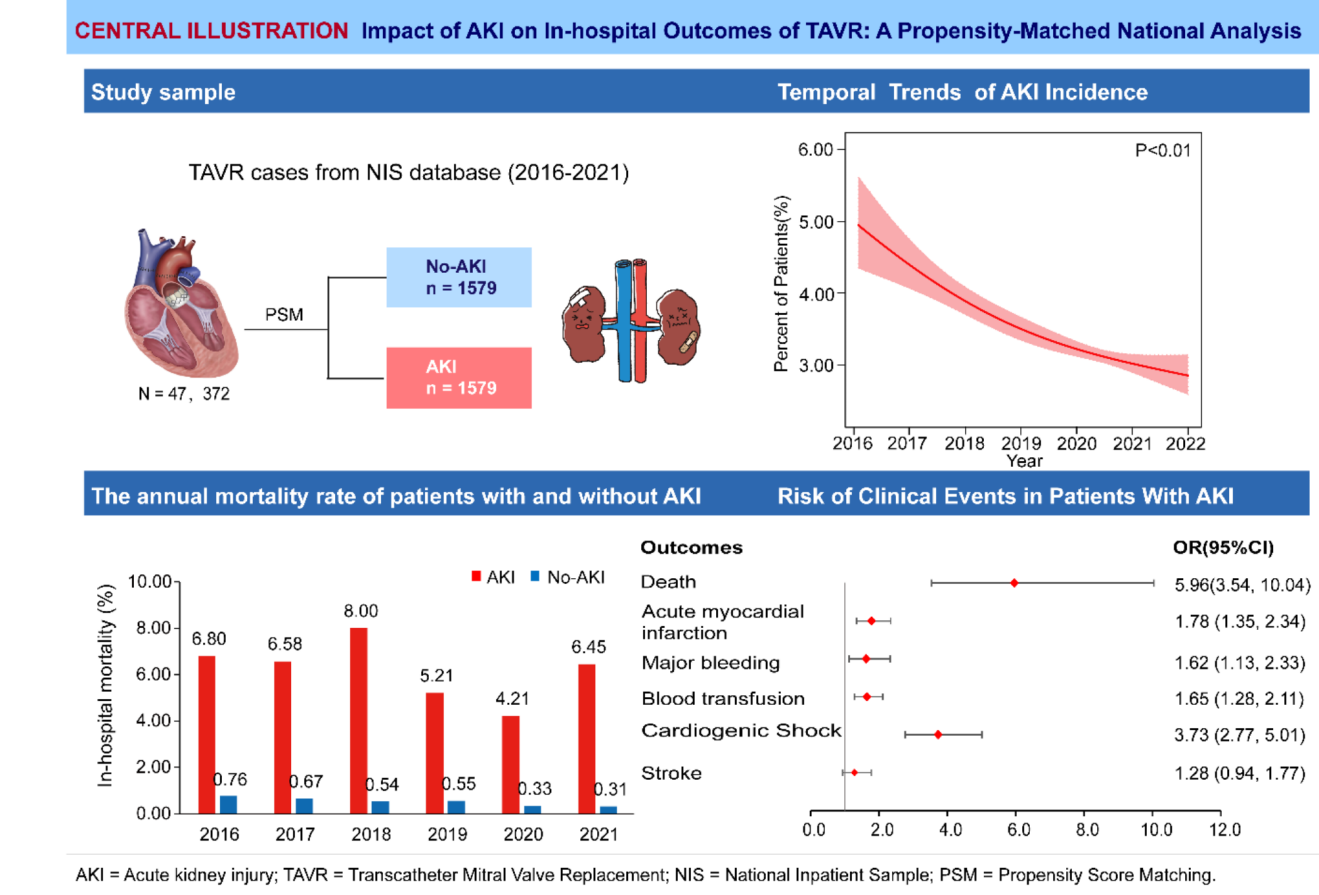
Key study findings

Among patients hospitalized with TAVR without CKD, the proportion of patients with AKI decreased from 4.82% in 2016 to 3.18% in 2021 (*P*-trend < 0.01) (shown in Fig. 4). In-hospital death remained high in patients with AKI, at 6.80% in 2016 and 6.45% in 2021 (*P*-trend = 0.51) (shown in Fig. 5).

## Discussion

In this large observational nationwide study of patients who had received TAVR with normal baseline renal function, we found several important findings. AKI was associated with an approximately 5-fold increase in in-hospital mortality. In patients with AKI, there was a significant increase in adverse in-hospital outcomes, including bleeding, blood transfusion, myocardial infarction, and CS. Moreover, while the rates of AKI incidence after TAVR declined over the study period, mortality rates were substantially higher in patients with AKI.

While AKI in TAVR-related hospitalized patients has been well investigated, there is insufficient information concerning AKI in the setting of patients without CKD. Recently, a registry study conducted in Finland by Moriyama et al. included retrospectively collected data from consecutive and unselected patients who had been treated with TAVR or surgical aortic valve replacement without CKD from 2008 to 2017. They reported that the overall incidence of AKI among patients receiving TAVR was 5.90% [23]. Our findings indicate that the incidence of AKI was lower (3.41%). Other studies with similar results have shown that the incidence of AKI after TAVR decreases to < 5% in patients with intermediate-to-low surgical risk and a low prevalence of CKD [11, 24, 25]. Several studies have reported an increased risk of mortality among a broad spectrum of hospitalized patients undergoing TAVR who develop AKI. Moriyama et al. showed that the risk of 5-year mortality increased 1.58-fold in patients with AKI [23]. Saia et al. reported that the development of AKI was associated with a doubling of mortality risk at 1-year follow-up (15.9% vs. 8.0%, respectively; *P* < 0.001), with a clear stepwise effect. The severity of the AKI stage had a negative effect on mortality [26]. The long-term effects of AKI have previously been investigated; however, studies evaluating the role of AKI on clinical outcomes in relation to patients who undergo TAVR without CKD have not been conducted. Differences in the effects of AKI on adverse outcomes may differ between regions and ethnic populations. Thus, the findings in this TAVR study may not be directly comparable to other related studies. We pooled all patients with AKI without a history of comorbid CKD (including stages 1 and higher) and found that AKI was associated with a 5.96 higher risk of mortality. Consistently, in a study using patients receiving TAVR alone derived from the NIS 2011–2014, Kumar et al. showed that AKI was associated with a significantly higher rate of mortality after TAVR (OR 6.58, 95% CI 5.25–8.24, *P* < 0.01) [27].

While studies have reported results concerning all-cause mortality, there is no conclusive evidence regarding other key clinical outcomes. We observed that AMI, CS, bleeding, and the need for transfusion were all significantly increased in patients with AKI, while the risk of stroke was increased but not significantly. A meta-analysis by Gargiulo et al. involving 5,971 patients treated with TAVR demonstrated that mortality, myocardial infarction, and blood transfusion were significantly increased in the AKI group, but no statistical difference was observed in relation to stroke [28]. Ma et al. reported that AKI increased the likelihood of early myocardial infarction, significant bleeding, and the requirement for a blood transfusion [29]. When comparing our results with those of Gargiolo et al. and Ma et al., certain differences in study design need to be considered. We excluded patients with CKD at baseline. With the continued advancement of TAVR technology, more accurate techniques in terms of annular size, and advances in procedural techniques, the incidence of AKI within our study population was likely to have been lower compared with the populations in those studies. Additionally, the burden of comorbidities among patients was lower in our cohort. Consequently, our findings indicate a reduced risk of the aforementioned adverse outcomes in comparison to the results reported in those studies. Our results complement those findings by utilizing a substantially larger sample size and contemporary data. Collectively, all these findings emphasize the elevated risk of complications associated with AKI.

There are several possible explanations for the associations between AKI and the identified adverse outcome risks post-TAVR. First, with regard to patient-related characteristics in this study, we observed that > 80% of hospitalized patients with TAVR and complicating AKI without any history of CKD had comorbid chronic heart failure. Several mechanisms exist that could explain the intricate bidirectional relationship between heart failure and the onset of AKI in hospitalized patients. Regarding the causes of renal insufficiency owing to heart failure, the most important pathophysiological mechanisms involved are neurohormonal activation, venous congestion, inflammation, effects of pharmacologic therapy for heart failure (renin-angiotensin -aldosterone system [RAAS] antagonists and diuretics), and nephrotoxic exposure [30]. In terms of AKI affecting heart failure and how it can lead to adverse outcomes, these involve abnormal electrolyte imbalances such as hyperkalemia that can cause arrhythmias, fluid overload that exacerbates congestive heart failure and pulmonary edema, as well as impaired cardiac contractility and response to catecholamines as a result of metabolic acidosis [31]. Additionally, patients with AKI had diabetes mellitus (DM) more frequently than those without AKI. DM is recognized as a significant predictor of contrast-induced nephropathy (CIN), as highlighted in the risk scores developed by Mehran et al. [32]. In patients with AKI, those who also have DM, especially with uncontrolled serum glucose levels, are likely to have diminished renal flow reserve. This reduction in flow reserve can exacerbate kidney injury and contribute to the overall severity of AKI. The presence of microvascular dysfunction in these patients is a critical factor that may worsen their prognosis. This dysfunction, often exacerbated by DM, emphasizes the intricate connection between metabolic control and renal health, particularly in the context of AKI [33]. Pre-procedural anemia has been identified as a predictor of AKI in TAVR and cardiac surgery [33, 34]. Some investigations have suggested that pre-operative anemia may harm the kidney directly or indirectly by raising patient vulnerability to concomitant renal insults (inflammatory response, renal hypoxia, and oxidative stress) [35]. However, the strength of the associations that remain after adjustment for important variables related to the severity of cardiovascular disease shows that these features may be confounding, while residual confounding cannot be ruled out. Additionally, our results show that patients with AKI had a greater risk of developing CS. CS is characterized by insufficient cardiac output, which leads to hypotension, and the kidneys, as an end organ, are directly affected by this hypoperfusion [22]. As a result, the combination of CS and AKI has a worse clinical scenario and a worse prognosis, with an increase in mortality and morbidity [36]. However, there was an increased risk of mortality among individuals who did not experience in-hospital shock, highlighting the effect of AKI even without this critical risk factor.

### Strengths and limitations

Our study has several strengths. This is the first study to examine the burden of AKI and its effect on adverse outcomes during hospitalization in patients who underwent TAVR without CKD in the United States. We used the most recent database, which is representative of real-world patients. Robust analyses were performed both before and after PSM, and subgroup analyses were included to investigate the consistency of associations across different patient subgroups. Multivariate regression and PSM models produced similar results, reflecting well on current practices and providing important comparative data to the current body of literature.

The present results, however, should be interpreted in light of some limitations. While utilization of the NIS provided an expansive database to analyze the incidence of AKI and its effect on clinical outcomes in patients who received TAVR over the most recent six years, data concerning some variables that might be important risk factors for clinical outcomes, such as the type, duration, timeline and severity of AKI, as well as its diagnosis (clinical diagnosis vs. laboratory-confirmed), contrast agent administration, type of anesthesia, hemodynamic parameters, types of valves (balloon-expandable or self-expandable), or other factors were not available. The NIS is reliant on ICD-10 codes, and there may have been coding and documentation errors. Nevertheless, the estimates, clinical characteristics, and procedural data from the NIS have been extensively validated internally and externally [37, 38]. Our data covered a period of six years, during which many changes and advances in the practice of TAVR occurred, both in the procedure itself and in patient selection and postprocedural care. Therefore, not all of the patients studied could be deemed representative of current clinical practice. Finally, this is an observational study with retrospective data, selection bias and unmeasured confounding factors cannot be avoided. Therefore, it is not appropriate to interpret the connection as causality.

## Conclusions

Our study demonstrated that AKI was associated with higher mortality and post-procedure complication rates among patients receiving TAVR without CKD. In-hospital outcomes were similar after PSM. Given that the benefits of TAVR are likely to expand indications for intervention to a larger population of patients at earlier stages of the disease, recognizing these risks and employing strategies to avoid AKI may improve post-TAVR outcomes.

## Electronic supplementary material

Below is the link to the electronic supplementary material.

Supplementary Material 1

## Data Availability

The data that support the findings of this study are openly available in The National (Nationwide) Inpatient Sample (NIS) at https://hcup-us.ahrq.gov/nisoverview.jsp.

## Abbreviations

AKI: Acute kidney injury
AMI: acute myocardial infarction
AS: aortic stenosis
CI: confidence interval
CIN: contrast-induced nephropathy
CKD: chronic kidney disease
CS: cardiogenic shock
DM: diabetes mellitus
ICD-10-CM/PCS: The International Classification of Diseases, Tenth Revision Clinical
IQR: interquartile range
KDIGO: the Kidney Disease Improving Global Outcomes
NIS: the National Inpatient Sample
OR: odds ratio
PSM: propensity score matching
SD: standard deviation
SMD: standardized mean difference
TAVR: transcatheter aortic valve replacement
