# Supplementary material for: Acute kidney injury and in-hospital outcomes after transcatheter aortic valve replacement in patients without chronic kidney disease: insights from the national inpatient sample
Source: BMC Cardiovasc Disord. 2024 Dec 19;24:706. doi: 10.1186/s12872-024-04303-1 (PMC11657798; doi:10.1186/s12872-024-04303-1)
Supplement: Supplementary file 1 — Supplementary Material 1 [file 12872_2024_4303_MOESM1_ESM.docx]

Table S1. ICD 10 diagnosis and procedure codes used in the study

| Elixhauser comorbidity | ICD-10 codes |
| --- | --- |
| Congestive Heart Failure | I09.9, I11.0, I13.0, I13.2, I25.5, I42.0, I42.5–I42.9, I43, I50, P29.0 |
| Cardiac Arrhythmia | I44.1–I44.3, I45.6, I45.9, I47–I49, R00.0, R00.1, R00.8, T82.1, Z45.0, Z95.0 |
| Valvular Disease | A52.0, I05–I08, I09.1, I09.8, I34–I39, Q23.0–Q23.3, Z95.2–Z95.4 |
| Pulmonary Circulation Disorders | I26, I27, I28.0, I28.8, I28.9 |
| Peripheral Vascular Disorders | I70, I71, I73.1, I73.8, I73.9, I77.1, I79.0, I79.2, K55.1, K55.8, K55.9, Z95.8, Z95.9 |
| Hypertension without Complications | I10 |
| Hypertension with Complications | I11–I13, I15 |
| Paralysis | G04.1, G11.4, G80.1, G80.2, G81, G82, G83.0–G83.4, G83.9 |
| Other Neurological Disorders | G10–G13, G20–G22, G25.4, G25.5, G31.2, G31.8, G31.9, G32, G35–G37, G40, G41, G93.1, G93.4, R47.0, R56 |
| Chronic Pulmonary Disease | I27.8, I27.9, J40–J47, J60–J67, J68.4, J70.1, J70.3 |
| Diabetes without Complications | E10.0, E10.1, E10.9, E11.0, E11.1, E11.9, E12.0, E12.1, E12.9, E13.0, E13.1, E13.9, E14.0, E14.1, E14.9 |
| Diabetes with Complications | E10.2–E10.8, E11.2–E11.8, E12.2–E12.8, E13.2–E13.8, E14.2–E14.8 |
| Hypothyroidism | E00–E03, E89.0 |
| Renal Failure | I12.0, I13.1, N18, N19, N25.0, Z49.0–Z49.2, Z94.0, Z99.2 |
| Liver Disease | B18, I85, I86.4, I98.2, K70, K71.1, K71.3–K71.5, K71.7, K72–K74, K76.0, K76.2–K76.9, Z94.4 |
| Peptic Ulcer Disease excluding Bleeding | K25.7, K25.9, K26.7, K26.9, K27.7, K27.9, K28.7, K28.9 |
| HIV/AIDS | B20–B22, B24 |
| Lymphoma | C81–C85, C88, C96, C90.0, C90.2 |
| Metastatic Cancer | C77–C80 |
| Solid Tumor without Metastasis | C00-C26, C30-C34, C37-C41, C43, C45-C58, C60- C76, C97 |
| Rheumatoid Arthritis/Collagen | L94.0, L94.1, L94.3, M05, M06, M08, M12.0, M12.3,  M30, M31.0–M31.3, M32–M35, M45, M46.1,  M46.8, M46.9 |
| Coagulopathy | D65–D68, D69.1, D69.3–D69.6 |
| Obesity | E66 |
| Weight Loss | E40–E46, R63.4, R64 |
| Fluid and Electrolyte Disorders | E22.2, E86, E87 |
| Blood Loss Anemia | D50.0 |
| Deficiency Anemia | D50.8, D50.9, D51–D53 |
| Alcohol Abuse | E52, F10, G62.1, I42.6, K29.2, K70.0, K70.3, K70.9, T51, Z50.2, Z71.4, Z72.1 |
| Drug Abuse | F11-F16, F18, F19, Z71.5, Z72.2 |
| Psychoses | F20, F22–F25, F28, F29, F30.2, F31.2, F31.5 |
| Depression | F20.4, F31.3–F31.5, F32, F33, F34.1, F41.2, F43.2 |
|  |  |
| Prior MI | I25.2 |
| Prior Stroke/TIA | Z86.73, I69 |
| Prior Cardiac arrest | Z86.74 |
| Prior PCI | Z98.61, Z95.5 |
| Prior CABG | Z95.1 |
| Prior pacemaker | Z950, |
| Prior Defibrillator | Z95.810 |
| Smoking | F17, T65, Z72.0, O99.33, Z87.891 |
| Dyslipidemia | E78X |
| Ischemic stroke | H34.1 I63.x I64.x |
| Hemorrhagic stroke | I60, I61, I629 |
| Transient ischemic attacks | G45.x |
| AKI | N17 N19 N99.89 |
| CABG | 0210, 0211, 0212, 0213 |
| PCI | 0270, 0271, 0272, 0273 |
| IABP | 5A02110, 5A02210 |
| Impella | 5A0221D，5A0211D |
| ECMO | 5A1522F, 5A1522G, 5A15A2F, 5A15A2G, 5A15223 |
| Permanent Pacemaker | 0JH604Z, 0JH634Z, 0JH804Z, 0JH834Z, 0JH605Z, 0JH635Z, 0JH805Z, 0JH835Z,  0JH606Z, 0JH636Z, 0JH806Z, 0JH836Z |
| STEMI | I21.x (except I21.4) |
| NSTEMI | I21.4 |
| Cardiogenic shock | R57, T8111XA |
| Procedural Hemorrhage | I97.410, I97.411, I97.418, I97.42, I97.610, I97.611, I97.618, I97.620 |
| Gastrointestinal Bleeding | K92.0-92.2; K25.0-25.2; K25.4-25.6; K26.0-26.2; K27.0-27.2; K27.4-27.6; K28.0-28.2; K28.4-28.6 |
| Intracranial Bleeding | I60-62 |
| Transfusion | 30233H0, 30233H1, 30233N0, 30233N1, 30233P0, 30233P1, 30230H0,30230H1, 30230N0, 30230N1, 30230P0, 30230P1, 30243H0, 30243H1,30243N0, 30243N1, 30243P0, 30243P1, 30240H0, 30240H1, 30240N0,30240N1, 30240P0, 30240P1 |
| Cardiac Arrest | I46.2, I46.8, I46.9 |
| CKD | N181, N182, N183, N184,N185, N189,N186 |
| Hemodialysis | T80.801, T80.902, T82.400, T82.401, Z99.201 |
| Peritoneal dialysis | T85.609, T85.610, T85.611, T85.710, T85.711, T85.801, T85.901, Z49.201 |
| TAVR | 02rf3*，02rf4* |

**TABLE S2 In-Hospital Outcomes of AKI vs. Non-AKI before Propensity-Score Matching.**

|  | Non-adjusted | | | Adjust-I | | | Adjust-I | | |
| --- | --- | --- | --- | --- | --- | --- | --- | --- | --- |
|  | OR | 95% CI | P-value | OR | 95% CI | P-value | OR | 95% CI | P-value |
| In-hospital mortality | 13.62 | (10.68, 17.37) | <0.01 | 9.95 | (7.40, 13.40) | <0.01 | 8.64 | (6.36, 11.74) | <0.01 |
| AMI | 8.06 | (6.71, 9.69) | <0.01 | 1.63 | (1.31, 2.03) | <0.01 | 1.67 | (1.34, 2.09) | <0.01 |
| Bleeding | 6.27 | (5.10, 7.71) | <0.01 | 1.75 | (1.36, 2.23) | <0.01 | 1.71 | (1.34, 2.20) | <0.01 |
| Blood transfusion | 5.07 | (4.36, 5.90) | <0.01 | 1.64 | (1.37, 1.96) | <0.01 | 1.60 | (1.34, 1.92) | <0.01 |
| CS | 4.99 | (3.63, 6.85) | <0.01 | 4.22 | (3.46, 5.15) | <0.01 | 4.14 | (3.37, 5.09) | <0.01 |
| Stroke | 3.81 | (3.06, 4.75) | <0.01 | 0.92 | (0.71, 1.18) | <0.01 | 0.87 | (0.67, 1.12) | <0.01 |

CI: Confidence interval, OR: Odds ratio.

Non-adjusted model adjusts for: No variates were adjusted.
Adjust I model: Adjusted for age; female; race; hospital division; median household income; cost of hospitalization; primary expected payer; length of stay.
Adjust II model: Adjusted for age; female sex; race; hospital division; median household income; cost of hospitalization; primary expected payer; length of stay; smoking; dyslipidemia; prior MI; prior stroke; prior cardiac arrest; prior PCI; prior CABG; and Elixhauser Comorbidity Index  .

**TABLE S3 In-Hospital Outcomes of AKI vs. Non-AKI after excluded CS patients in the Propensity-Score Matched Cohort**

|  | OR | 95% CI | P-value |
| --- | --- | --- | --- |
| In-hospital mortality | 4.45 | (2.32, 8.57) | <0.01 |
| AMI | 2.02 | (1.43, 2.86) | <0.01 |
| Bleeding | 1.35 | (0.85, 2.15) | 0.19 |
| Blood transfusion | 1.57 | (1.16, 2.12) | <0.01 |
| Stroke | 1.51 | (1.03, 2.22) | 0.03 |
